# A Perfect Absorber Based on Similar Fabry-Perot Four-Band in the Visible Range

**DOI:** 10.3390/nano10030488

**Published:** 2020-03-08

**Authors:** Pinghui Wu, Congfen Zhang, Yijun Tang, Bin Liu, Li Lv

**Affiliations:** 1 Research Center for Photonic Technology, Fujian Key Laboratory for Advanced Micro-nano Photonics Technology and Devices & Key Laboratory of Information Functional Material for Fujian Higher Education, Quanzhou Normal University, Quanzhou 362000, China; phwu@zju.edu.cn; 2 College of Life Science and Engineering, Southwest University of Science and Technology, Mianyang 621010, China; Zhang2017@swust.edu.cn; 3 College of Science, Zhejiang University of Technology, Hangzhou 310023, China; tyj1970@zjut.edu.cn; 4 School of Physics and Electric Engineering, Linyi University, Linyi 276000, China; liubin@lyu.edu.cn

**Keywords:** broadband, metamaterial, perfect absorber, four-band absorption, COMSOL, surface plasmon polariton

## Abstract

A simple metamaterial absorber is proposed to achieve near-perfect absorption in visible and near-infrared wavelengths. The absorber is composed of metal-dielectric-metal (MIM) three-layer structure. The materials of these three-layer structures are Au, SiO_2_, and Au. The top metal structure of the absorber is composed of hollow three-dimensional metal rings regularly arranged periodically. The results show that the high absorption efficiency at a specific wavelength is mainly due to the resonance of the Fabry–Perot effect (FP) in the intermediate layer of the dielectric medium, resulting in the resonance light being trapped in the middle layer, thus improving the absorption efficiency. The almost perfect multiband absorption, which is independent of polarization angle and insensitivity of incident angle, lends the absorber great application prospects for filtering and optoelectronics.

## 1. Introduction

The dielectric constant of metal materials is less than zero in visible and infrared bands, which is contrary to the symbol of the dielectric constant. When micro and nanoscale metals and dielectrics are combined and interact with the electromagnetic field, they can produce different or even opposite optical phenomena in nature, such as negative refraction and slow light effect [1,2,3,4,5]. In recent years, it has been found that the combination of metal and dielectric materials can transform the incident electromagnetic wave into surface plasmon polariton (SPP), to enhance the light regulation ability of materials at the nanoscale [6]. Usually, when the light in free space travels to the air–metal interface, most of the energy is reflected, and some of it is converted into heat loss and absorbed by the material. Only a little power can penetrate the metal. However, with the appearance of metal-dielectric micro/nano structures, we can realize enhanced resonance absorption of electromagnetic waves in specific wavebands using surface iso-excimer resonance or other resonance effects. Among these structures, metal-dielectric-metal-based multilayer structures have natural magnetic resonance properties. They can respond to the incident magnetic field and generate antiparallel current on two metal surfaces. Thus, a local electromagnetic field is generated in the medium, which enhances the absorption of electromagnetic waves by the metal. On the macroscopic scale, a nanophotonic device with full absorption at a specific wavelength is formed. This device is an electromagnetic absorber [7].

When a material is used primarily to absorb certain bands of electromagnetic waves, we call it an electromagnetic absorber. Electromagnetic absorbers can be used in many different fields according to their absorption band, absorption capacity, absorption principle, and absorption spectrum [8,9,10,11,12,13,14]. For example, the Salisbury screen is mainly used in the military to reduce the reflection of radar detectors by canceling the interference of reflected microwaves by the reflection layer [15]. The same principle can be used in optical frequencies to design an anti-reflective film on a camera lens or glass. For another example, a pyramid-shaped structure can increase the number of reflections and scatter in the structure by matching the impedance of free space, thus increasing the absorption efficiency [16].

Since the concept of the perfect absorber was introduced in 2008, the research on superstructure electromagnetic material absorbers has shown exponential growth year by year [17,18,19,20]. From the initial microwave frequency band to terahertz [20,21], infrared [22,23], and visible frequency band [24,25,26,27], a large number of studies have been conducted, and absorption bandwidth also includes single-frequency absorption, dual-frequency absorption, multi-frequency absorption, and broadband absorption [28,29,30,31,32,33]. Compared with the traditional absorption materials, the advantage of the superstructure material absorber lies in the structure size, arrangement, and different materials selected to form the dual-frequency, multi-frequency, or dual-band absorber [34,35,36]. The structure size is small and the processing method is simple, which is conducive to the integration in the period surface [37,38,39]. The structure or quantity can be flexibly adjusted to achieve different absorption purposes. Due to the variety of absorbers, the absorbers have a different applicable wavelengths, bandwidths, structure characteristics, adjustable abilities, and absorption principles. In this paper, a multi-band metamaterial absorber in the visible light range is designed from the perspective of the absorption frequency band. By changing the structure size of the absorber and the metal structure on the surface, the absorber can achieve a broader tolerance range for polarization and incidence angle, which has potential for application in filtering and optoelectronics.

## 2. Physical Design

**Figure 1:**
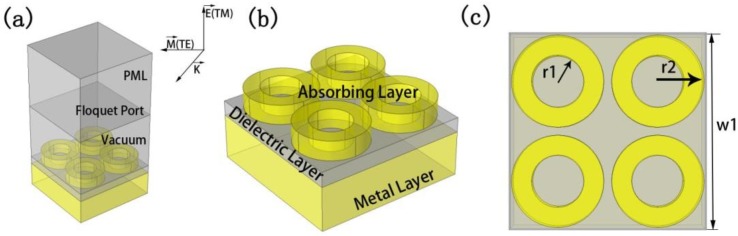
Basic absorber schematic: (**a**) a periodic unit structure of the absorber, (**b**) schematic diagram of the components of the absorber, and (**c**) top view of the absorber.

The metamaterial absorber we designed is shown in Figure 1. It has a metal-dielectric-metal multilayer structure from the bottom to the top. For the bottom layer of metal, we used gold as the material and as a metal mirror; the thickness was 300 nm. The intermediate dielectric layer was SiO_2_. The depth of SiO_2_ was 100 nm and the relative dielectric constant was 2.13 [40,41]. The absorption layer was composed of a three-dimensional hollow metal ring arranged in a periodic structure. When constructing the geometric model, in order to find the best value, we used different values of 80-120 nm for the thickness of the node layer. We also changed the spacing between the hollow solid rings. At the same time, to study the relationship between the material of the absorption layer and the absorption, we chose different materials as the absorption layer for simulation. Its optimal structure size and parameters were: model width w_1_ = 1000 nm, three-dimensional circle inner diameter r_1_ = 230 nm, and three-dimensional circle inner diameter r_2_ = 130 nm. The thickness of the reflective metal layer was h_1_ = 300 nm. The thickness of the dielectric layer was h_2_ = 100 nm. The thickness of the absorption layer gold was h_3_ = 130 nm and the ring spacing of the absorption layer was d = 40 nm. We used the Drude model to set the detailed parameters of Au and commercial software COMSOL to simulate the structure [42].

The fabrication method of the structure is simple in practical application. First, an Au array was prepared by electron beam evaporation. Then, high-resolution electron beam (EB) lithography and reactive ion coating method were used to regulate the viscosity, and the rotation speed of the coating method was used to regulate the viscosity and rotation speed of the coating material to accurately control the thickness of SiO_2_ layer. Finally, the gold array was prepared again using electron beam evaporation and ultraviolet lithography [43,44].

During the simulation with COMSOL, by setting the boundary conditions and port types of the metamaterial absorber, the metamaterial absorber we designed can become a periodic array structure. In a simulation, the absorption of the metamaterial absorber can be expressed as A(ω) = 1 − R(ω) − T(ω) [45,46,47,48,49]. A(ω) is the absorptivity, R(ω) is reflectivity and T(ω) is transmittance. However, the incorporation of the metamaterial absorber can be rewritten as A(ω) = 1 − R(ω), if the transmitted light is zero due to the presence of the metal mirror in the absorber. In addition, although the MIM (metal-dielectric-metal) structure has three layers, generally only a two-layer model can represent the three-layer MIM structure. Because the MIM structure at the bottom of the metal layer is often add after, it can simulate semi-infinite metal substrate, leading to almost zero transmission energy. The relative permeability is 1, the metal-dielectric substrate (top half infinite) relative dielectric constant is n_1_, which a negative real part, and the imaginary part is zero. The dielectric layer in the middle of the relative dielectric constant is n_2_, with a positive real part, and the imaginary part is not null. Therefore, when the light in the absorber is incident, it will have a resonance coupling effect with the dielectric layer and the absorption layer, causing the light to be absorbed by the absorber. The absorption intensity is also related to the relative permittivity of the metal and the dielectric layer, and the absorption can be adjusted through the relative permittivity.

## 3. Results and Discussions

In the absorber, the top structure corresponds to a Fabry-Perot cavity. The absorption principle is based on interference enhancement and interference reduction at the interface. The electromagnetic field is reflected and transmitted multiple times on the interface in the metamaterial, and finally superimposed multiple times in the incident plane. If the superimposed result is zero or small, it means that the electromagnetic field has formed interference cancellation in the incident surface after passing through the metamaterial, so there is no reflection or the reflection is very small. Because the back is metal, the electromagnetic field cannot pass through, so the electromagnetic field is completely absorbed. Hence, the electromagnetic field is totally absorbed, which explains the absorption principle of the metamaterials. Therefore, the resonant frequency and absorption efficiency of the absorber can be changed by changing the geometric and structural parameters of the absorber.

**A. The study of different polarization patterns**

**Figure 2:**
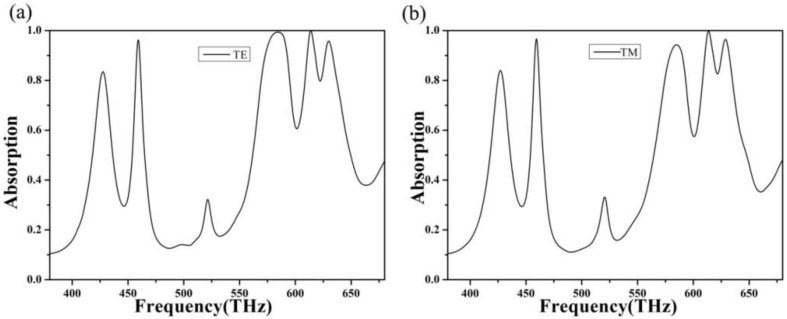
(**a**) The absorption curve under TE polarization, and (**b**) the absorption curve under TM polarization.

**Figure 3:**
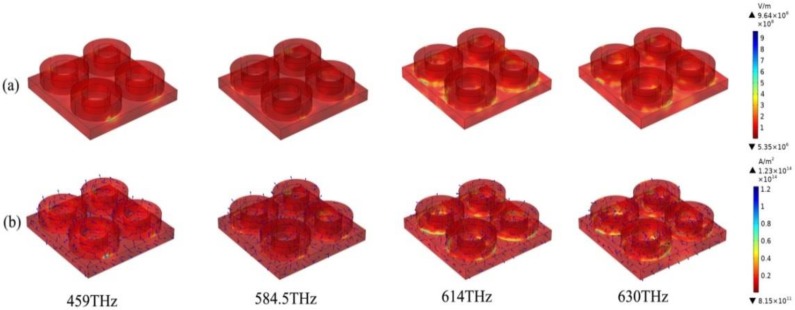
(**a**,**b**) The electric field and current distribution of the absorber in four resonant modes: 459, 584.5, 614, and 630 THz.

Figure 2 shows the absorption curve of the absorber under the polarization of TE (transverse electric, the electric field is parallel to the Y direction) and TM (transverse magnetic, the electric field is parallel to the X direction). By observing Figure 2, we can find that the absorber has four resonance absorption modes, among which there are three resonance absorption peaks at 459, 614, and 630 THz, with the absorption of 96.2%, 99.9%, and 95.8%, respectively. A broadband absorption was also produced in the 523–592.5 THz range with more than 90% absorption, and broadband center absorption reached 99.3%. In addition, by comparing Figure 2a with Figure 2b, we see that the position and intensity of resonance absorption of the absorber are approximately the same, whether in TE mode or TM mode. This is because the absorption layer of the absorber is arranged periodically and regularly. Regardless of incidence from the X direction or event from the Y direction, it has excellent light absorption characteristics. To further study the multipeak absorption of the absorber, we drew the electric field current scanning diagram at different peak times, as shown in Figure 3.

Figure 3 shows the electric field current diagram of the absorber. We show the electric field and surface current density of the absorber in different resonance modes in the figure, and draw the direction of the electromagnetic field. In Figure 3, by observing the electric fields at 459 and 584.5 THz, only a small part of the layer has a coupling resonance with the node layer. At 630 and 614 THz, all absorbing and dielectric layers showed resonance coupling. Electromagnetic field energy was a local area in the middle of the dielectric layer. We drew the electric displacement vector of cloth as well as the direction, which clearly shows the lower level metal surface had opposite surface currents, formed in the middle of the SiO_2_ circuit, and a negative magnetic response in the positive direction. However, in TM mode, different polarization states were caused due to different incident directions, thus slightly deviating the absorption curve.

**B. Study on absorption of different structural parameters**

**Figure 4:**
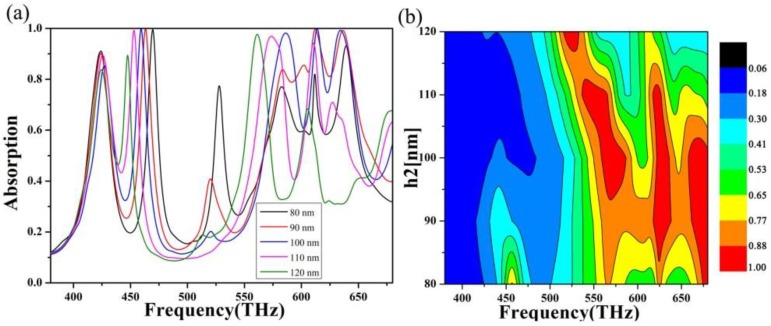
(**a**) Absorption curve and (**b**) absorption images of scanning dielectric layer thickness (80–120 nm) at 380–680 THz.

First, we studied the effect of the absorber on the absorption when the thickness of the dielectric layer is changed. In this study, we used a controlled variable approach to ensure that the content under review was not affected by other parameters of the absorber. The thickness of the dielectric layer was taken from nm to 120 nm with a step length of 10 nm, and the scanning absorption image in the visible band was drawn, as shown in Figure 4. By looking at the absorption lines, we can see that the absorption summit moves toward the lower frequency side as the dielectric layer thickness increases. Redshift can be explained by the propagation phase, where α is the propagation phase, which is caused by:

(1) $$\alpha =\frac{4h\sqrt{\varepsilon_{r}-\sin^{2}\theta }}{\lambda },$$

where h is the thickness of the dielectric layer, ϵ is the real part of the effective dielectric constant of the absorber, θ is the incident angle, and λ is the terahertz wavelength. Since ϵ and θ are attached, if  α is considered a fixed value, then h/λ is also fixed. This means that h is inversely proportional. Therefore, as the thickness of the dielectric layer increases, the center frequency will shift to the lower frequency. We can also find that the four absorption peaks, on the whole, show a trend of first increasing and then decreasing [50,51,52]. At the dielectric layer thickness of 100 nm, high peak values can be guaranteed at the four resonance positions. Therefore, at this time, the surface impedance of the absorber is perfectly matched with the free space impedance, achieving the best absorption effect.

**Figure 5:**
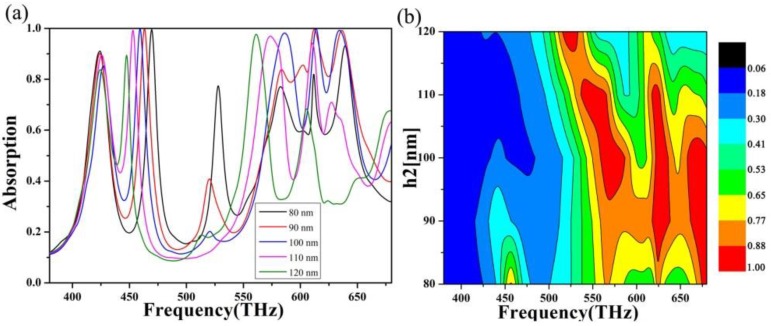
The absorption images of the three-dimensional ring pitch (30–50 nm) of the absorption layer when the frequency is changed from 380–680 THz (**a**,**b**).

Secondly, we studied the effect of changing the spacing of the four hollow solid rings in the absorption layer of the absorber by using the control variable method. In this study, the spacing of the three-dimensional hollow ring was evaluated from 30 to 50 nm with a step length of 5 nm, and the scanning absorption image in the visible light band was drawn, as shown in Figure 5. By observing Figure 5, we can find that when the spacing changes, the position of the absorption peak will not change, but the intensity will vary. This is because when the distance between the absorption layer changes, the resonance intensity between the absorption layer and the dielectric layer below the absorber changes [53,54]. Besides, the height of the absorption layer determines the position of the absorption peak.

**Figure 6:**
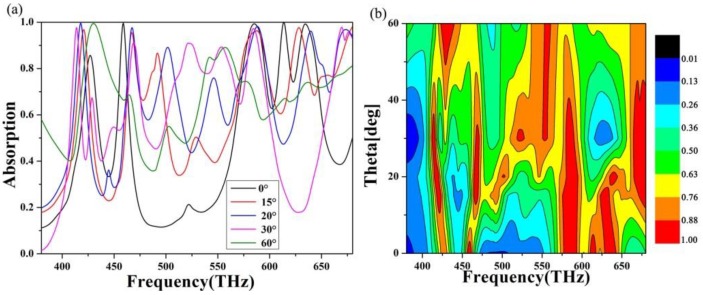
The absorption images at scanning incidence angle (0°–60°) at 380–680 THz (**a**,**b**).

In addition, considering the practical application of the absorption layer, we simulated the absorption of the absorber at a large angle and small angle, as shown in Figure 6. It can be seen from the figure that when the perspective of the absorber changes in a small area, the absorption change of the absorber is small. When the angle of incidence is large, the overall absorption of the absorber is significantly reduced, but there are still perfect absorption peaks. When the incident angle is zero, the absorption effect is better, there are three absorption peaks, and the existing absorption bandwidth is also extensive. As the aspect of incidence changes, the position of the absorption peak and the intensity of the absorption changes. When the incident angle of the absorber increases, the absorption rate decreases due to the increase in light reflection under high oblique incidence. In addition, when the incident angle increases, the magnetic dipole oscillation of the entire vibration absorber is effectively excited so that the strong absorption characteristics are maintained at a large angle, which is also the reason why there is a perfect absorption peak at a large angle. In general, the absorber can operate within and maintain a very high ideal absorption regardless of whether the incident angle is small or large.

**C. Effects of different material properties on absorber absorption**

As different metal materials correspond to different plasma and collision frequencies, the absorption performance of the absorber will also alter. Therefore, we selected four common metal materials, namely gold, silver, aluminum, and copper, to simulate the absorption performance of the absorber.

**Figure 7:**
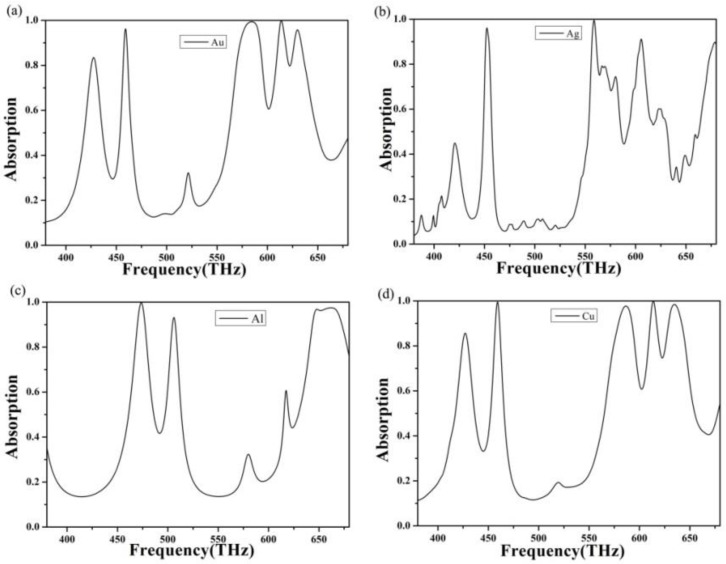
Change in the absorption spectrum of the absorber metamaterial. (**a**) The absorption curve when the material is gold, (**b**) the absorption curve when the material is silver, (**c**) the absorption curve is when the material is aluminum, and (**d**) the absorption curve when the material is copper.

The absorption properties of gold were studied in detail in the previous part of this paper, so we will not elaborate this part in detail. As the material properties of gold and silver are similar, we first selected Ag as the metal mirror of the absorber and the top absorption structure layer [55,56]. The absorption curve is shown in Figure 7b. We can observe through observation that when silver is used as the material of the absorption layer, there are only two resonance absorption peaks with an absorption rate of more than 90%. Besides, the absorption curve wave is relatively apparent. There are a lot of low absorption peaks due to the incident light irradiation on the silver and the following SiO_2_. Different frequencies of light will make the two parts produce resonance a coupling effect. Also, the absorption layer can absorb different frequencies of incident light, which caused some messy small peaks to appear. At the same time, the positions of the two stiff coupling peaks are changed due to different material properties.

Considering that the price of gold and silver is generally higher and the actual processing cost is higher, we think that Al and Cu, given their lower prices, should be used as the material for the metal layer of the absorber. We changed the absorption layer of the absorber and the content at the bottom into aluminum; the resulting absorption curve is shown in Figure 7c. Through observation, we found that when Al was used as the material of absorption layer, the absorption curve changed considerably compared with that of gold, the position of resonance absorption peak shifted, and the intensity of absorption peak also significantly improved. Broad-spectrum absorption occurs in the range of 644 and 670 THz [57,58]. This is because the plasma frequency of Al is higher than that of gold, which leads to a change in the resonant coupling mode between the absorption layer and the dielectric layer, resulting in wide-spectrum absorption. In addition, the position of resonance absorption peak changed due to the change in plasma frequency.

Finally, the most common metal material Cu was considered and simulated, as shown in Figure 7d. Through observation, we found that the absorption curve of copper was almost completely coincident with that of gold because the plasma frequency and collision frequency of copper are very close to that of gold. Therefore, when visible light shone on it, the resonance coupling mode was almost the same. Compared with copper, metallography has a broadband absorption in the absorption curve, which is more favorable for practical applications such as filter detection.

## 4. Conclusions

In conclusion, we designed a simple visible metamaterial perfect absorber with three classic absorption peaks and small broadband. The intensity of absorption peaks and broadband intensity can reach above 97.8% on average. We studied the absorption curves of the absorber under different polarization conditions and found that the absorber has polarization insensitivity. We also studied the polarization angle, incident angle, and other parameters of the absorber in detail, indicating that the absorber can work stably under different polarizations and incident angles. In addition, the absorption characteristics of different materials of the absorber were studied, and we was found that the absorber can maintain good absorption characteristics when the material of the absorption layer is Au. The above characteristics make the absorber have potential for application in the fields of filtering and photoelectricity.
